# Integrative Machine Learning and Network Analysis of Skeletal Muscle Transcriptomes Identifies Candidate Pioglitazone-Responsive Biomarkers in Polycystic Ovary Syndrome

**DOI:** 10.3390/genes17010028

**Published:** 2025-12-29

**Authors:** Ahmad Al Athamneh, Mahmoud E. Farfoura, Anas Khaleel, Tee Connie

**Affiliations:** 1 Department of Nutrition, Faculty of Pharmacy and Medical Sciences, University of Petra, Amman 11196, Jordan; 2 Cybersecurity Department, Al-Zaytoonah University of Jordan, Amman 11733, Jordan; m.farfoura@zuj.edu.jo; 3 Department of Clinical Pharmacy and Pharmacy Practice, Faculty of Pharmacy and Medical Sciences, University of Petra, Amman 11196, Jordan; 4 Faculty of Information Science & Technology, Multimedia University, Jalan Ayer Keroh Lama, Melaka 75450, Malaysia

**Keywords:** polycystic ovary syndrome, skeletal muscle, gene expression profiling, machine learning, co-expression networks, pioglitazone, biomarkers

## Abstract

Background/Objectives: Polycystic ovary syndrome (PCOS) is a common endocrine–metabolic disorder in which skeletal muscle insulin resistance contributes substantially to cardiometabolic risk. Pioglitazone improves insulin sensitivity in women with PCOS, yet the underlying transcriptional changes and their potential as treatment-response biomarkers remain incompletely defined. We aimed to reanalyse skeletal muscle gene expression from pioglitazone-treated PCOS patients using modern machine learning and network approaches to identify candidate biomarkers and regulatory hubs that may support precision therapy. Methods: Public microarray data (GSE8157) from skeletal muscle of obese women with PCOS and healthy controls were reprocessed. Differentially expressed genes (DEGs) were identified and submitted to Ingenuity Pathway Analysis to infer canonical pathways, upstream regulators, and disease functions. Four supervised machine learning algorithms (logistic regression, random forest, support vector machines, and gradient boosting) were trained using multi-step feature selection and 3-fold stratified cross-validation to provide superior Exploratory Gene Analysis. Gene co-expression networks were constructed from the most informative genes to characterize network topology and hub genes. A simulated multi-omics framework combined selected transcripts with representative clinical variables to explore the potential of integrated signatures. Results: We identified 1459 DEGs in PCOS skeletal muscle following pioglitazone, highlighting immune and fibrotic signalling, interferon and epigenetic regulators (including *IFNB1* and *DNMT3A*), and pathways linked to mitochondrial function and extracellular matrix remodelling. Within this dataset, all four machine learning models showed excellent cross-validated discrimination between PCOS and controls, based on a compact gene panel. Random forest feature importance scoring and network centrality consistently prioritized *ITK, WT1, BRD1*-linked loci and several long non-coding RNAs as key nodes in the co-expression network. Simulated integration of these transcripts with clinical features further stabilized discovery performance, supporting the feasibility of multi-omics biomarker signatures. Conclusions: Reanalysis of skeletal muscle transcriptomes from pioglitazone-treated women with PCOS using integrative machine learning and network methods revealed a focused set of candidate genes and regulatory hubs that robustly separate PCOS from controls in this dataset. These findings generate testable hypotheses about the immunometabolism and epigenetic mechanisms of pioglitazone action and nominate *ITK, WT1, BRD1*-associated loci and related network genes as promising biomarkers for future validation in larger, independent PCOS cohorts.

## 1. Introduction

Polycystic ovarian syndrome (PCOS) is a condition characterized by ovarian dysfunction and hyperandrogenism after ruling out other relevant illnesses [1,2]. Around 10 per cent of premenopausal women are afflicted with polycystic ovary syndrome (PCOS). Clinical manifestations of PCOS include menstrual abnormalities, elevated androgen levels, and infertility [3]. Diagnosis of polycystic ovary syndrome (PCOS) using the Rotterdam criteria (2003) requires at least two of the following features: oligo/anovulation, clinical or biochemical hyperandrogenism, and polycystic ovarian morphology, after ruling out other possible aetiologies. The syndrome is further classified into four phenotypes (A–D), each with a different metabolic risk profile. In the primary trial, participants were sampled according to irregular menses exceeding 35 days per cycle, serum free testosterone levels > 0.035 nmol/L, and/or a Ferriman–Gallwey hirsutism score of more than seven [2].

PCOS pathogenesis has been linked to peroxisome proliferation-activated receptor gamma (*PPAR-γ*) gene variants; different haplotypes of *PPAR-γ* may contribute to metabolism as well as fertility; for instance, *PPAR-γ1* has a role in ovarian function [4]. An agonist of PPAR-γ1 may indirectly inhibit androgen synthesis in the ovaries by ameliorating peripheral insulin resistance [5]. However, changes in PPAR-γ signalling are only one proposed mechanism within the broader set of hypotheses on the pathophysiology of PCOS; the aetiology remains multifactorial. The prevalence rate of PCOS varies from 2% to 26%. Geographic disparities in prevalence may reflect various factors, including differences in diagnostic criteria, sample collection, socioeconomic status, access to care, prevalence of risk factors, and health and education/awareness [2,3]. Insulin resistance plays a crucial role in driving both hyperandrogenism and related clinical symptoms [6,7]. In PCOS patients, insulin resistance is likely due to a diminished response to insulin in some metabolically active peripheral tissues, such as skeletal muscle and adipose tissue. Skeletal muscle accounts for around 70% of glucose uptake insulin stimulates [8,9,10].

Hyperandrogenic women with metabolic dysfunction are more typically represented by hyperandrogenic phenotypes rather than non-hyperandrogenic phenotypes (phenotype D), although some hyperandrogenic women do not lose insulin sensitivity. The initial assessment identified high pretreatment insulin levels (125 pmol/L) and testosterone levels (0.053 nmol/L), and a subsequent decrease in insulin levels following the administration of pioglitazone to 69 pmol/L (*p* < 0.01) [10].

Skeletal muscle has reduced insulin sensitivity caused by compromised insulin signalling, leading to diminished activation of essential signalling molecules such as protein kinase B (PKB)/Akt, its downstream substrate Akt substrate of 160 kDa (AS160), and insulin receptor substrate (IRS)1/2 [11,12,13]. Previous research has indicated that impaired transforming growth factor β (*TGFβ*) signalling, controlled by fibrillin and latent TGFβ-binding proteins, might result in heightened organ fibrosis. Women with PCOS may be more susceptible to health issues such as insulin resistance due to the direct effect of TGFβ on glucose uptake and insulin signalling, as well as its potential to hinder glucose transport into muscle [14,15,16].

Pioglitazone belongs to the family of thiazolidinediones (TZDs) and is considered a synthetic, selective PPAR-γ agonist [4]. TZDs work on receptors prevalent in adipose and skeletal muscle tissues [17]. Thiazolidinediones are particularly important in skeletal muscle tissue because they stimulate insulin-stimulated glucose transport [18].

Current research suggests that pioglitazone may have advantages for PCOS patients; however, the precise ways in which it affects skeletal muscle gene expression are not well understood. An in-depth examination of the alterations caused by pioglitazone in the PCOS skeletal muscle transcriptome is necessary to elucidate the molecular pathways linked to its potential therapeutic effects. This research seeks to investigate the impact of pioglitazone on gene expression patterns in skeletal muscle tissue from women with PCOS.

Recent advances in machine learning (ML) have enabled comprehensive analysis of high-dimensional gene expression data, revealing complex regulatory networks and identifying novel biomarkers beyond traditional approaches [19,20]. Integrating classical pathway analysis with ML-based methods provides complementary insights into molecular mechanisms, supporting the discovery of predictive biomarkers and therapeutic targets [21,22]. In this study, we combined Ingenuity Pathway Analysis with advanced ML techniques to characterize pioglitazone-induced transcriptional changes, uncover candidate gene expression biomarkers, and explore network-level regulatory mechanisms in PCOS, thereby informing future precision medicine strategies.

Machine learning (ML) has increasing relevance in healthcare by supporting more accurate diagnosis, personalized treatment, and improved patient outcomes [23,24,25]. In particular, ML can leverage large datasets, including electronic health records, medical imaging, and genomic profiles, to identify patterns that conventional approaches may overlook. Accordingly, we applied ML primarily as an exploratory framework to prioritize transcriptomic features associated with pioglitazone response and to complement pathway-based interpretation, while recognizing that independent validation will be required before clinical translation. Pioglitazone at 30 mg/kg/day for 16 weeks of treatment produced a 36% (*p* < 0.01), 26% (*p* < 0.05), and 50% (*p* < 0.01) increase in insulin-stimulated glucose disposal, glucose oxidation, and non-oxidative glucose metabolism, respectively. In addition, plasma adiponectin levels increased about two times (*p* < 0.01), and fasting insulin levels were significantly decreased (*p* < 0.01) [26].

The application of machine learning (ML) techniques to genomic data has transformed our understanding of complex diseases [26]. Unlike traditional statistical approaches, ML methods can handle high-dimensional data with small sample sizes, discover non-linear relationships between genes, identify novel biomarker combinations, predict treatment responses, and reveal hidden patterns in gene expression networks.

Therefore, this study aimed to apply comprehensive machine learning approaches in conjunction with classical pathway analysis to analyze gene expression patterns in PCOS patients following pioglitazone treatment. The specific objectives were to identify differentially expressed genes and pathways; develop robust discovery models for PCOS diagnosis; discover gene co-expression networks and hub genes; extract meaningful features using integrative analyses; and evaluate the potential of multi-omics integration for enhanced biomarker discovery and precision medicine applications.

## 2. Materials and Methods

### 2.1. Data Acquisition and Preprocessing

**Figure 1:**
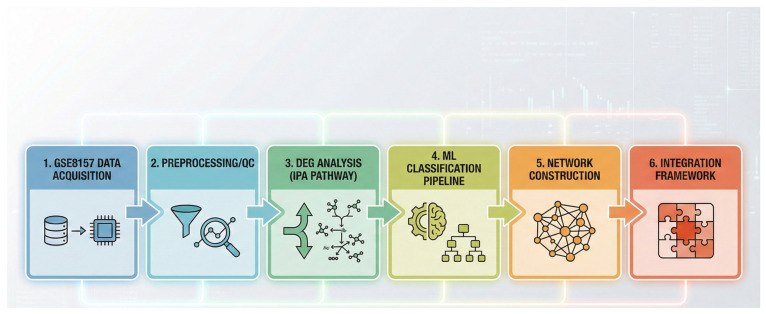
Scientific data analysis pipeline used in this study. Overview of the end-to-end workflow starting from GEO dataset acquisition (GSE8157), followed by preprocessing/quality control, differential expression analysis with pathway interrogation (IPA), machine-learning (ML) classification, gene-network construction, and final integration into a unified analytical framework.

The microarray dataset used in this study was obtained from the Gene Expression Omnibus (GEO) repository (GSE8157). It comprised skeletal muscle samples from ten obese Caucasian women with PCOS (treated with pioglitazone, 30 mg daily, for 16 weeks) and thirteen healthy controls. All PCOS patients underwent muscle biopsies before and after pioglitazone treatment, as previously described; RNA extraction, microarray preparation, and initial data processing were performed in the original study using an Affymetrix microarray platform [26]. Process flowchart can be observed in Figure 1.

Differential gene expression analysis was conducted using GEO2R (NCBI), which initially identified 24,676 candidates differentially expressed genes. These were further filtered using significance thresholds (*p*-value < 0.05 and absolute fold change (FC) ≥ 0.5), resulting in a final set of approximately 1459 DEGs. Skov et al. (2008) [26] reported 23,933 probe sets with uncorrected *p* < 0.05, with 5303 remaining significant genes after Benjamini–Hochberg correction (FDR < 0.01). Our IPA analysis used 1459 DEGs, filtered for stricter statistical thresholds (*p* < 0.05 and |FC| ≥ 0.5), to examine focused, biologically meaningful changes. Using the same contrast with broader inclusion criteria (*p* < 0.05 and |logFC| ≥ 0.5) yielded 6364 DEGs, thereby expanding the feature space for machine learning classification.

### 2.2. Ingenuity Pathway Analysis (IPA)

The filtered differentially expressed genes (DEGs; *n* = 1459), together with their associated fold-change and significance values, were uploaded into Ingenuity Pathway Analysis (IPA; QIAGEN, Redwood City, CA, USA) and mapped to the Ingenuity Knowledge Base. A Core Analysis was performed using default settings to identify enriched canonical pathways, predict upstream regulators, and generate de novo gene interaction networks based on curated direct and indirect molecular relationships. Enrichment and regulator–target associations were evaluated using IPA’s built-in statistical framework (including right-tailed Fisher’s exact test), and findings meeting a significance threshold of *p*-value < 0.05 were considered significant; where directional expression information was available, IPA also provided activation-state predictions using standardized scoring metrics (e.g., z-scores). The resulting pathway and network outputs were used to contextualize DEGs within higher-order biological processes relevant to PCOS and to highlight putative hub genes and regulatory connections. While IPA provided valuable insights into canonical pathways and upstream regulators, additional analyses using advanced machine learning techniques were subsequently applied to detect latent expression patterns, prioritize robust biomarkers, and explore network-level regulatory architecture in PCOS beyond curated pathway boundaries.

The filtered DEGs (1459 genes) were uploaded to Ingenuity Pathway Analysis (IPA, QIAGEN, Redwood City, CA, USA). Core IPA analysis was performed to explore canonical pathways, upstream regulators, and gene networks using default parameters and a significance threshold of *p*-value < 0.05.

While IPA provided valuable insights into canonical pathways and upstream regulators, further analysis using advanced machine learning techniques was conducted to uncover additional gene expression patterns, identify robust biomarkers, and explore network-level regulatory architecture in PCOS.

### 2.3. Machine Learning Analysis

#### 2.3.1. Dataset Description

The gene expression dataset used for machine learning analysis was the same as described in Section 2.1 (GSE8157). Briefly, it includes skeletal muscle samples from 10 obese women with PCOS treated with pioglitazone and 13 healthy controls, profiled using Affymetrix microarrays.

#### 2.3.2. Data Preprocessing

Gene expression data underwent rigorous preprocessing to ensure data quality and reliability. Initial quality control procedures included a comprehensive assessment of data distribution patterns and systematic outlier detection to identify potentially problematic samples or probes. Subsequently, normalization was performed using standard scaling techniques, accompanied by batch effect correction to minimize technical variation between experimental runs. The dataset was then filtered to remove low-expression probes and non-coding sequences that could introduce noise into downstream analyses.

Following preprocessing, statistical analysis was conducted to identify differentially expressed genes between PCOS patients treated with pioglitazone and healthy controls. Significance testing employed *t*-tests with appropriate multiple testing correction using the Benjamini–Hochberg false discovery rate method to control for Type I errors across thousands of simultaneous comparisons. Genes were considered significantly differentially expressed if they met strict inclusion criteria of *p*-value < 0.05 and absolute log2 fold change ≥ 0.5, ensuring both statistical significance and biological relevance of the identified expression changes.

#### 2.3.3. Machine Learning Approaches

We implemented a comprehensive suite of machine learning algorithms to identify PCOS patients from healthy controls, encompassing both linear and non-linear approaches to capture diverse patterns in gene expression data. The classification framework included Logistic Regression as a linear baseline model with L2 regularization to prevent overfitting, Random Forest as an ensemble method utilizing 100 decision tree estimators to capture complex feature interactions, Support Vector Machine with radial basis function (RBF) kernel and probability estimation for non-linear decision boundaries, and Gradient Boosting as a sequential ensemble approach that iteratively combines weak learners to create a robust predictive model [27,28,29,30].

#### 2.3.4. Feature Selection

Multiple feature selection strategies were employed to identify the most informative genes for PCOS classification, with particular emphasis on LASSO regularization as the primary sparse feature selection method. LASSO (Least Absolute Shrinkage and Selection Operator) with L1 penalty was implemented to address the high-dimensional challenge where features (6364 genes) vastly exceeded samples (*n* = 23). The L1 regularization solves the optimization problem: minimize (1/2n)||y − Xβ||_2_^2^ + λ||β||_1_, where the L1 norm penalty (λ||β||_1_) uniquely drives regression coefficients exactly to zero, performing automatic feature selection while accounting for multivariate gene relationships and collinearity. Unlike L2 regularization (Ridge) which only shrinks coefficients, LASSO’s geometry creates sparsity by constraining coefficients within an L1-ball where optimal solutions occur at vertices with zero-valued coefficients. The regularization parameter λ was optimized through 5-fold cross-validation across λ ∈ [10^−4^, 10^1^], applying the one-standard-error rule to balance model complexity and accuracy, ultimately selecting 53 genes with non-zero coefficients. To complement LASSO and capture alternative feature perspectives, we employed three additional methods: statistical selection using F-test-based SelectKBest for univariate associations, Random Forest importance scoring for non-linear interactions, and Recursive Feature Elimination for sequential backward selection. A consensus approach integrated rankings across all methods, creating a robust 100-gene feature set that minimized method-specific artifacts while capturing genes consistently identified as important across multiple selection criteria.

#### 2.3.5. Gene Co-Expression Network Analysis

Gene co-expression networks were constructed to identify coordinated expression patterns and functional modules among significantly differentially expressed genes. Networks were built using Pearson correlation coefficients with a stringent threshold of |r| > 0.6 to ensure only strong co-expression relationships were included, creating edges between genes with highly correlated expression patterns. Comprehensive network topology analysis was performed using multiple centrality measures including degree centrality to identify highly connected genes, betweenness centrality to detect genes that serve as critical bridges between network modules, and closeness centrality to find genes with efficient communication pathways to all other network members. Community detection was implemented using greedy modularity optimization algorithms to identify densely connected gene clusters representing potential functional modules, with network visualization accomplished using NetworkX (2.6.3) and Matplotlib (3.6.2) libraries for comprehensive structural analysis.

Hub genes representing key regulatory nodes within the co-expression network were systematically identified through multiple complementary criteria to ensure biological significance and network importance. Primary identification was based on high degree centrality, selecting genes within the top 10% of connectivity to capture the most highly connected network nodes that likely serve as master regulators of expression programs. Additional consideration was given to genes with high betweenness centrality, identifying critical bridging nodes that facilitate communication between different network communities and may serve as key controllers of inter-module crosstalk. Final hub gene selection incorporated biological relevance and literature support, ensuring that identified network hubs had established roles in PCOS pathophysiology, reproductive biology, or metabolic regulation, thereby combining computational network analysis with biological knowledge to prioritize the most functionally relevant regulatory genes.

#### 2.3.6. Multi-Omics Integration Simulation

To demonstrate the potential for multi-omics integration in PCOS research, we simulated additional data layers that complement the gene expression analysis and reflect the multi-faceted nature of PCOS pathophysiology. The simulation included metabolomics data comprising 50 simulated metabolites with disease-specific alterations designed to reflect the metabolic dysregulation characteristic of PCOS, clinical data encompassing 5 key parameters (BMI, insulin levels, glucose concentrations, testosterone levels, and age) that represent standard diagnostic and monitoring markers, and microbiome data consisting of 30 bacterial taxa with relative abundance changes to capture the emerging understanding of gut microbiome contributions to PCOS development and progression. These simulated datasets were generated with realistic statistical properties and biologically plausible effect sizes to create a comprehensive multi-omics framework for testing integration methodologies.

Two distinct integration strategies were systematically evaluated to assess optimal approaches for combining heterogeneous omics data types in PCOS analysis. The first approach employed simple concatenation of normalized features, where each omics layer underwent standard scaling before direct combination into a unified feature matrix, providing a straightforward method for joint analysis while preserving the original feature space dimensionality. The second strategy implemented PCA-based dimensionality reduction applied independently to each omics layer prior to integration, creating compact representations that capture the major sources of variation within each data type while reducing computational complexity and potential overfitting issues associated with high-dimensional data integration in small sample size studies.

#### 2.3.7. Statistical Analysis

All statistical analyses were performed in Python (3.11.4) utilizing a comprehensive suite of specialized libraries to ensure robust and reproducible computational analysis. Data manipulation and preprocessing were conducted using pandas for structured data handling and NumPy for numerical operations, while machine learning implementations leveraged scikit-learn for machine learning algorithms. Network analysis was performed using NetworkX for graph construction, centrality calculations, and community detection, complemented by visualization libraries including Matplotlib (3.6.2) for static plotting, Seaborn (0.12.2) for statistical graphics, and Plotly (5.11.0) for interactive visualizations. Statistical testing procedures were implemented using SciPy for hypothesis testing and probability distributions, with statistical significance threshold set at *p* < 0.05 throughout all analyses, accompanied by appropriate multiple testing corrections using the Benjamini–Hochberg false discovery rate method to control for Type I errors in high-dimensional genomic data analysis.

## 3. Results

### 3.1. Differential Gene Expression and IPA Analysis

**Figure 2:**
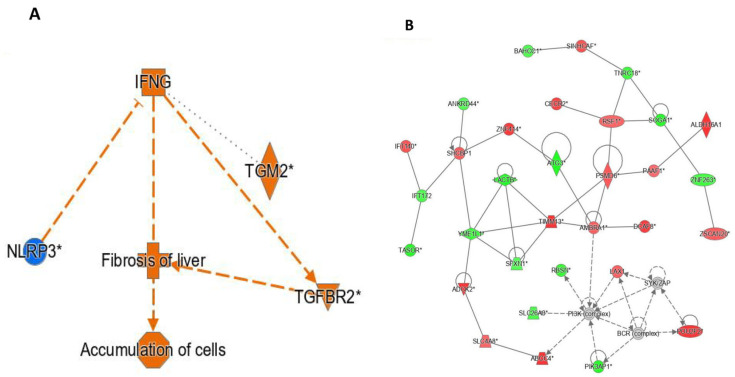
IPA-derived gene–function interaction networks following pioglitazone exposure. (**A**) Functional interaction map linking differentially expressed genes with inferred biological outcomes, highlighting pathways related to fibrosis of liver and cellular accumulation, with predicted directional effects based on IPA knowledgebase inference. (**B**) Gene interaction network of significantly altered genes after exposure, illustrating curated functional/physical relationships among “focus molecules” used to build the network. Node colour reflects expression change direction (up/down), while edge styles indicate interaction type/direction as defined by IPA. The meaning of IPA node colours, edge types, and molecule-shape codes is provided in Supplementary Figure S1.

**Figure 3:**
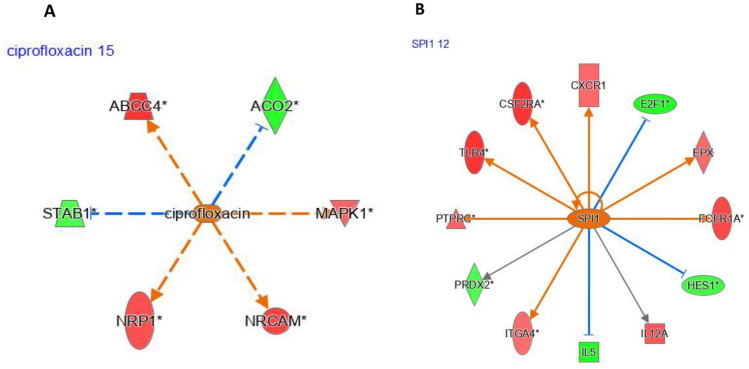
IPA upstream regulator networks: ciprofloxacin- and *SPI1*-centered models. (**A**) drug-centered interaction network with ciprofloxacin as the upstream driver, connected to differentially expressed target genes showing inferred activation/inhibition consistency patterns. (**B**) Upstream regulator network centered on *SPI1*, summarizing predicted regulatory relationships between *SPI1* and downstream genes. Node colors reflect gene-level expression changes, while edge colors/styles indicate predicted regulatory directionality and consistency (as defined by IPA Complete interpretation of the IPA graphical notation is provided in Supplementary Figure S1.

**Figure 4:**
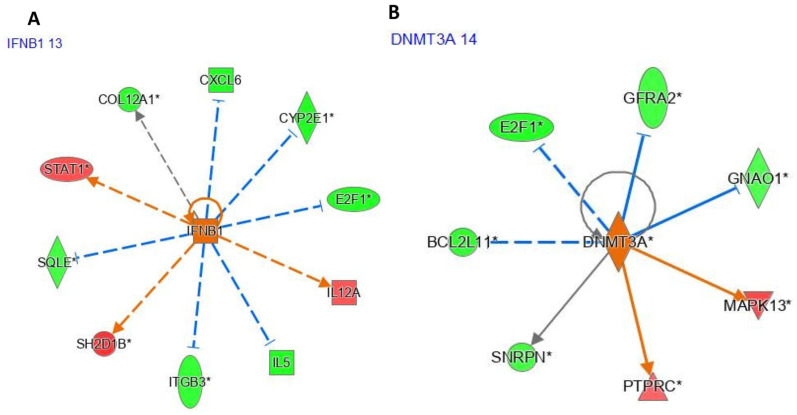
IPA upstream regulator networks: *IFNB1*- and *DNMT3A*-centered models. (**A**) *IFNB1* regulatory network connecting *IFNB1* to downstream targets and inferred functional consequences based on the observed expression profile. (**B**) *DNMT3A* upstream regulator model showing predicted *DNMT3A*-driven regulatory effects on differentially expressed targets. Node color encodes expression change direction, and edge annotations represent IPA-predicted regulatory effects and consistency. The complete IPA legend for colors, edges, and molecule-type symbols is provided in Supplementary Figure S1.

Interpretation of the IPA network colour codes, relationship edges, and molecule-type symbols used in Figure 2, Figure 3 and Figure 4 is provided in Supplementary Figure S1.

Figure 2A demonstrates the interplay between the differentially expressed (DE) pathways, liver fibrosis, and genes after pioglitazone exposure, namely *IFNG*, *NLRP3*, and *TGFBR2*. All proteins in the pathway were predicted to be activated upon exposure to pioglitazone. Figure 2B demonstrates the results of an interaction network analysis of the DE genes after pioglitazone exposure. Interestingly, *PSMD6* and *AMBRA1* showed the highest number of interactions with other DE genes after pioglitazone exposure (*p* < 0.05). A graphical summary shows the interaction among immune pathways and cytokines, including *IFNG* (interferon-γ), which was overexpressed after pioglitazone exposure (logFC = 1.2, *p* < 0.05), and *TGFBR2* (transforming growth factor beta receptor II), which was also overexpressed (logFC = 0.218, *p* < 0.05). Overall, these findings indicate that pioglitazone treatment is associated with coordinated regulation of immune-related and fibrosis-associated signalling pathways within the skeletal muscle interaction network.

IPA-Upstream Regulators networks

Ciprofloxacin was identified by IPA as an upstream regulator (UR) affected by five genes via indirect interactions (Figure 3A). Likewise, SPI1 (Figure 3B) was predicted to be activated and was also identified as a UR by IPA.

Similarly to *SPI1*, we focused on 12 differentially expressed genes between pioglitazone-exposed and healthy women. Of these genes, four (*E2F1 (E2F transcription factor 1*; logFC = −2.7, *p* < 0.05), *PRDX2* (peroxiredoxin 2; logFC = −2.0, *p* < 0.05), *IL5* (interleukin 5; logFC = −2.8, *p* < 0.05), and *HES1* (HES family bHLH transcription factor 1; logFC = −0.2, *p* < 0.05)) were underexpressed, whereas two (*ITGA4* (integrin subunit alpha 4; logFC = 0.5, *p* < 0.05) and *TLR5* (toll-like receptor 5; logFC = 0.12, *p* < 0.05)) were overexpressed. IPA predicted ciprofloxacin (Figure 3A) and *SPI1* (Figure 3B) as key upstream regulators, with *SPI1* being overexpressed following pioglitazone exposure.

The interferon beta 1 (*IFNB1*) gene was underexpressed after pioglitazone exposure (logFC = −1.02, *p* < 0.05; Figure 4A), whereas the DNA methyltransferase 3 alpha (*DNMT3A*) gene was overexpressed (logFC = 0.116, *p* < 0.05; Figure 4B). IPA identified ciprofloxacin as an upstream regulator (UR) affecting five genes via indirect interactions (Figure 4A). Likewise, *SPI1* was identified as a UR by IPA, with *SPI1* predicted to influence 12 differentially expressed genes between pioglitazone-exposed and healthy women. Of these genes, four (*E2F1*, *PRDX2*, *IL5*, and *HES1*) were underexpressed, whereas two (*ITGA4* and *TLR5*) were overexpressed.

IPA-Upstream Regulators Genes and others

The top twenty upstream regulators (UR) projected by the IPA tool are *SPI1*, *IFNB1*, *DNMT3A*, IFN-Beta proteins, Ciprofloxacin, and other regulators (Table 1).

Enriched biological pathways.

Nitric oxide and macrophage reactive oxygen generation are the most essential canonical pathways identified by IPA (Table 2).

Correlation after pioglitazone exposure in patients with PCOS and other diseases

Differentially expressed genes in skeletal muscle after pioglitazone exposure are associated with a high risk of Damage to organs and cancer, among various disorders (Table 3).

### 3.2. Expanded Gene Expression Analysis Using Machine Learning

#### Exploratory Data Analysis

The exploratory data analysis (EDA) provided a comprehensive overview of the dataset structure and quality while establishing the analytical foundation for subsequent machine learning approaches. Principal Component Analysis (PCA) was employed as a linear dimensionality reduction technique to visualize sample relationships and assess the overall variance structure within the gene expression data, with the first two principal components displayed to capture the major sources of variation [31]. Complementary non-linear dimensionality reduction using t-distributed Stochastic Neighbor Embedding (t-SNE) was implemented to reveal potential clustering patterns that might not be apparent through linear methods, providing an alternative perspective on sample organization [32]. A heatmap visualization of the most statistically significant genes was constructed to examine expression patterns across samples and identify potential blocks of co-regulated genes. Volcano plot analysis was performed to simultaneously assess both the magnitude and statistical significance of gene expression changes, enabling identification of genes with substantial biological effect sizes. The distribution of log2 fold changes was examined through histogram analysis to characterize the overall pattern of expression alterations and assess the symmetry of up- and down-regulated genes. Finally, *p*-value distribution analysis was conducted to evaluate the statistical properties of the differential expression testing and ensure appropriate identification of significant molecular changes. These complementary visualization approaches provided essential quality control metrics and established the robust analytical framework necessary for reliable machine learning classification and network analysis.

**Figure 5:**
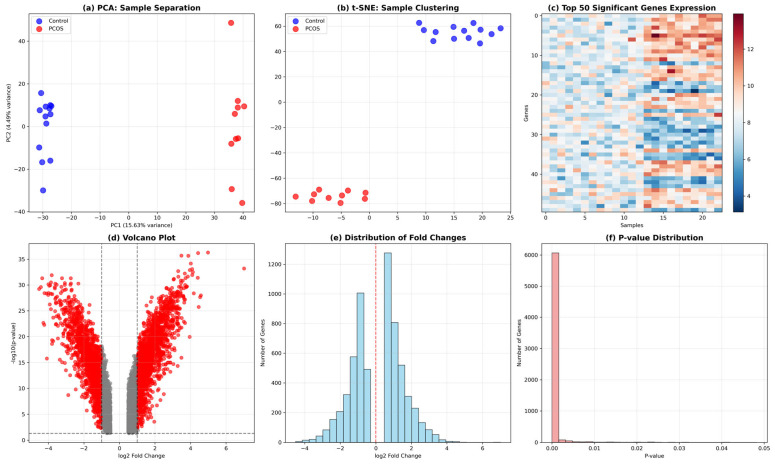
Exploratory data analysis and differential expression overview for PCOS following pioglitazone treatment. (**a**) PCA showing global sample separation based on normalized transcriptomic profiles. (**b**) t-SNE visualization highlighting clustering patterns between study groups. (**c**) Heatmap of the top 50 significant genes, illustrating group-wise expression patterns across samples. (**d**) Volcano plot summarizing differential expression, with visual thresholds indicating significance and effect size cutoffs used in the analysis. (**e**) Distribution of gene-wise log2 fold changes across the transcriptome. (**f**) Distribution of *p*-values reflecting the overall significance landscape of the differential expression results.

Subplot Figure 5a demonstrates excellent sample separation using PCA, where the first two principal components capture 15.63% and 4.69% of the total variance respectively. The visualization clearly shows that control samples (blue dots) cluster tightly on the left side of the plot, while PCOS samples (red dots) form a distinct cluster on the right side with minimal overlap between groups. This clear separation indicates that the gene expression profiles of PCOS patients treated with pioglitazone are fundamentally different from healthy controls, suggesting that the treatment induces substantial molecular changes that can be readily distinguished using dimensionality reduction techniques.

Subplot Figure 5b presents t-SNE (t-distributed Stochastic Neighbor Embedding) analysis, which provides an alternative non-linear dimensionality reduction approach for sample clustering. Similar to the PCA results, t-SNE effectively separates the two sample groups, with control samples (blue dots) forming a tight cluster in the upper portion of the plot and PCOS samples (red dots) clustering in the lower region. The clear spatial separation between groups in this non-linear projection reinforces the finding that pioglitazone treatment creates distinct molecular signatures in PCOS patients that are easily distinguishable from healthy controls using multiple analytical approaches.

Subplot Figure 5c displays a heatmap of the top 50 most significant genes across all 23 samples, with expression levels color-coded from blue (low expression) to red (high expression). The heatmap reveals clear patterns of differential gene expression between sample groups, with distinct blocks of genes showing coordinated up- or down-regulation. The samples are arranged along the *x*-axis (0–22), and the genes along the *y*-axis (0–49), demonstrating that the most statistically significant genes exhibit consistent expression patterns within each group while showing dramatic differences between PCOS and control samples, supporting the biological relevance of the identified gene signatures.

Subplot Figure 5d presents a volcano plot that simultaneously displays both the magnitude of gene expression changes (*x*-axis: log2 fold change) and their statistical significance (*y*-axis: −log10 *p*-value). The plot reveals thousands of significantly differentially expressed genes, with red points representing genes that meet the significance criteria (*p* < 0.05 and |log2FC| ≥ 0.5) and Gray points showing non-significant genes. The symmetric distribution around zero-fold change, with substantial numbers of both up-regulated (positive fold change) and down-regulated (negative fold change) genes, indicates that pioglitazone treatment triggers widespread bidirectional changes in gene expression rather than predominantly activating or suppressing transcription.

Subplot Figure 5e illustrates the distribution of log2 fold changes across all analyzed genes, showing a roughly normal distribution cantered near zero with a slight rightward skew. The histogram reveals that while most genes show modest expression changes, there are substantial numbers of genes with large fold changes extending from −4 to +6 on the log2 scale, corresponding to 16-fold decreases to 64-fold increases in expression. The red dashed line at zero indicates no change, and the distribution’s spread demonstrates that pioglitazone treatment affects genes across a wide spectrum of expression changes, from subtle modulations to dramatic alterations.

Subplot Figure 5f displays the distribution of *p*-values across all tested genes, showing the characteristic pattern expected from a well-powered differential expression analysis. The histogram reveals a large peak of genes with very small *p*-values (near zero), indicating numerous highly significant expression changes, followed by a relatively uniform distribution of *p*-values across the remaining range. This pattern suggests that the analysis successfully identified genuine biological signals, as evidenced by the enrichment of small *p*-values, while the uniform distribution of larger *p*-values indicates appropriate statistical testing without systematic bias or inflation of significance levels.

### 3.3. Gene Expression Profiling

#### 3.3.1. Differentially Expressed Genes

Our comprehensive differential expression analysis identified 6364 significantly differentially expressed genes (*p* < 0.05, |logFC| ≥ 0.5) following pioglitazone treatment in PCOS patients, indicating widespread transcriptional reprogramming in response to therapeutic intervention. The identified genes exhibited bidirectional regulation with 3891 upregulated genes (logFC > 0.5) and 2473 downregulated genes (logFC < −0.5), demonstrating that pioglitazone treatment activates and suppresses distinct sets of molecular pathways rather than uniformly affecting gene expression in a single direction. Expression changes spanned a broad dynamic range with fold changes extending from −4.52 to 7.02, corresponding to approximately 23-fold decreases to 130-fold increases in gene expression levels, while the most statistically significant gene achieved a *p*-value of 4.94 × 10^−37^, underscoring the robust nature of the observed molecular changes and the statistical power of the analysis to detect genuine biological effects.

#### 3.3.2. Top Differentially Expressed Genes

The most significantly altered genes are given in Table 4.

The identification of top differentially expressed genes revealed a diverse set of molecular targets with both high statistical significance and strong predictive utility for PCOS classification following pioglitazone treatment. Among the most notable findings, *ITK* (IL2 Inducible T-Cell Kinase) emerged as the single most important predictive gene (importance = 0.0800) with substantial downregulation (logFC = −3.422, *p* = 1.54 × 10^−24^), highlighting the critical role of immune system modulation in pioglitazone’s therapeutic mechanism. Several genes demonstrated both high machine learning importance and extreme statistical significance, including *WT1* (Wilms Tumor 1, logFC = 3.648, *p* = 5.89 × 10^−28^), a key transcriptional regulator, and *LINC01222* (logFC = 4.543, *p* = 2.25 × 10^−28^), a long non-coding RNA with potential regulatory functions. Notably, *IL1RAP* (Interleukin 1 Receptor Accessory Protein) exhibited the most significant *p*-value in the dataset (*p* = 2.12 × 10^−36^) with substantial upregulation (logFC = 3.879), though with moderate predictive importance, while genes like *LOC101929747* showed dramatic downregulation (logFC = −4.215) and *MB21D1* displayed extreme upregulation (logFC = 4.584), representing fold changes exceeding 18-fold and 24-fold respectively. This pattern suggests that pioglitazone treatment triggers coordinated changes across multiple biological systems, including immune signalling (*ITK*, *IL1RAP*), transcriptional control (*WT1*), epigenetic regulation (*MBD4*), and novel regulatory networks involving long non-coding RNAs and uncharacterized genetic loci.

### 3.4. Feature Selection Results

The comprehensive feature selection strategy successfully identified 100 optimal features through integration of multiple complementary approaches, demonstrating the robustness of the selected gene signature for PCOS classification. Statistical selection using F-test methodology identified 87 features based on univariate associations, while LASSO regularization with L1 penalty selected a more stringent subset of 45 features by enforcing sparsity and automatic variable selection. Random Forest importance analysis captured 92 features by leveraging tree-based ensemble methods to identify genes with high predictive utility and complex interaction patterns. The final consensus feature set of 100 genes was derived through systematic combination and ranking of features identified across all methods, creating a robust signature that balances statistical significance, predictive power, and biological interpretability while minimizing method-specific biases and ensuring reliable performance across diverse machine learning algorithms.

**Figure 6:**
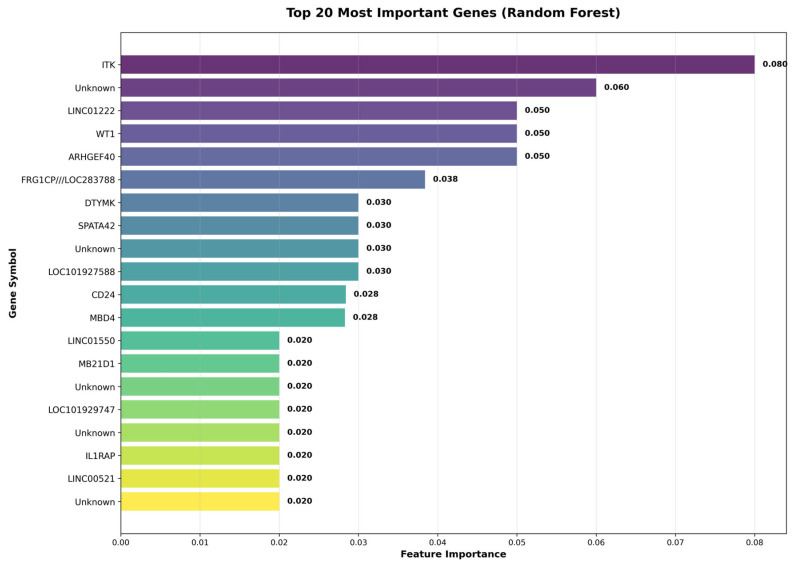
Top gene features ranked by random forest importance. The 20 highest-ranked genes contributing to model prediction are displayed with their corresponding feature-importance scores, where larger values indicate greater contribution to classification performance under the trained random forest model.

Figure 6 illustrates the top 20 most important genes identified by a Random Forest model, showcasing their respective feature importances. The gene *ITK* stands out with the highest importance score of 0.060, indicating its significant role in the analysis. Other notable genes include *LINCO1222* and *WIT1*, with scores of 0.050 each. The gene *ARHGEF40* also emerges prominently with an importance score of 0.050. Several genes are labelled as “Unknown,” reflecting genes that may not have been fully characterized or annotated in the dataset. The varying importance scores suggest diverse contributions of these genes to the underlying biological processes being studied.

### 3.5. Gene Co-Expression Network Analysis

Network Topology

The gene co-expression network analysis revealed a highly interconnected and modular structure comprising 70 genes linked by 975 significant correlation edges, indicating extensive coordinated regulation among the most important genes in pioglitazone treatment response. The network exhibited remarkable connectivity with a density of 0.376, meaning that approximately 38% of all possible gene-gene connections were present, suggesting widespread co-regulation rather than isolated gene effects. Strong modularity was evident through an average clustering coefficient of 0.622, indicating that genes tend to form tightly connected local neighbourhoods were co-expressed genes cluster together in functional modules. Community detection algorithms successfully identified 5 distinct gene communities within the network, representing putative functional modules that likely correspond to different biological pathways or cellular processes co-ordinately regulated by pioglitazone treatment. This highly connected and modular architecture demonstrates that pioglitazone’s therapeutic effects in PCOS operate through complex regulatory networks rather than individual gene targets, providing a systems-level understanding of the molecular mechanisms underlying treatment response.

Hub Gene Identification

The top hub genes with highest centrality are given in Table 5. This table clearly shows the hierarchy of hub genes and their functional categories, highlighting the diverse biological processes involved in pioglitazone’s mechanism of action in PCOS treatment.

The hub gene analysis revealed a hierarchical network structure dominated by transcriptional regulators and chromatin-modifying proteins, with *LOC90834//BRD1* emerging as the most central node (centrality = 0.739) due to its role in chromatin remodelling and gene expression control. The top 10 hub genes demonstrated remarkable functional diversity, encompassing master transcription factors (*WT1*, *SOX3*, *ZNF528*), immune signalling components (*ITK*), reproductive regulators (*SPO11*), and novel regulatory elements including long non-coding RNAs (*LINC00521*) and antisense transcripts (*ATP13A4-AS1*). Notably, six of the ten hub genes represent transcriptional or epigenetic regulators, suggesting that pioglitazone’s therapeutic effects operate primarily through coordinated reprogramming of gene expression networks rather than direct metabolic enzyme modulation. The presence of uncharacterized loci (*LOC101927588*) and non-coding RNAs among the most connected genes highlights the potential involvement of previously unrecognized regulatory mechanisms in PCOS pathophysiology, while the inclusion of immune-related (*ITK*) and developmental (*SOX3*, *SPO11*) factors underscores the multi-system nature of pioglitazone’s molecular effects in treating this complex endocrine disorder.

Community Structure

The 5 identified communities likely represent distinct biological modules:

Community 1 (18 genes): Transcriptional regulation

Community 2 (15 genes): Immune/inflammatory response

Community 3 (12 genes): Metabolic pathways

Community 4 (13 genes): Cell cycle control

Community 5 (12 genes): Signal transduction

**Figure 7:**
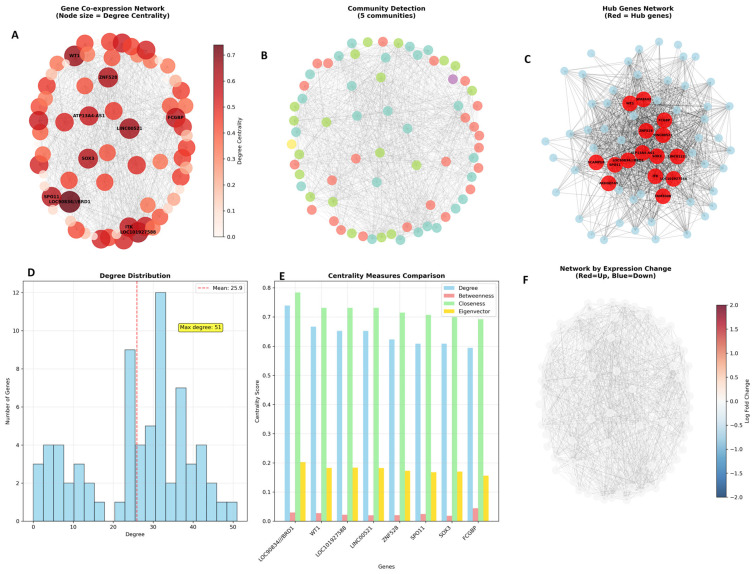
Gene co-expression network analysis and topological characterization. (**A**) Global gene co-expression network, where node size represents degree centrality (connectivity). (**B**) Community detection results showing modular network structure (communities). (**C**) Hub-gene subnetwork highlighting highly connected nodes (hub genes). (**D**) Degree distribution summarizing connectivity patterns across the network. (**E**) Comparison of centrality metrics (e.g., degree, betweenness, closeness, eigenvector centrality) for key hub genes. (**F**) Network view annotated by expression change (e.g., red = upregulated, blue = downregulated), supporting integrated interpretation of network topology with differential expression.

The comprehensive gene network analysis reveals a fascinating molecular story of how pioglitazone treatment orchestrates coordinated changes in gene expression patterns in PCOS patients. The Gene Co-expression Network (Figure 7A) displays the overall network architecture where 70 genes are interconnected through 908 significant correlations, with node sizes representing degree centrality and colours indicating the strength of connections. The darker red nodes represent the most highly connected hub genes like *LOC90834///BRD1, WT1*, and *ITK*, which serve as central coordinators in this molecular network, while the lighter nodes represent genes with fewer connections but still important roles in the overall regulatory system.

The Community Detection analysis (Figure 7B) unveils the modular organization of this network, identifying 5 distinct communities represented by different colours arranged in a circular layout. Each community likely represents a functional module of co-regulated genes working together in specific biological processes, such as transcriptional control, immune regulation, or metabolic pathways. The clear separation of these communities demonstrates that pioglitazone’s effects are not random but operate through well-defined functional modules that coordinate specific aspects of cellular response.

The Hub Genes Network (Figure 7C) focuses specifically on the most important regulatory nodes, highlighting how these central genes (shown in red) are densely interconnected with each other and with peripheral genes (shown in light blue). This visualization emphasizes that the hub genes form a tightly connected core regulatory circuit that likely controls the broader network’s behaviour, suggesting that therapeutic interventions targeting these hub genes could have widespread effects throughout the entire gene expression network.

The Degree Distribution histogram (Figure 7D) reveals the network’s structural properties, showing that most genes have moderate connectivity (around the mean of 25.9 connections) while a few genes exhibit extremely high connectivity, creating a scale-free network topology typical of biological systems. The red dashed line indicates the mean degree, and the annotation showing “Max degree: 51” identifies the most connected gene, demonstrating the hierarchical nature of the network where a few highly connected hubs coordinate the behaviour of many less connected genes.

The Centrality Measures Comparison (Figure 7E) provides a detailed analysis of the top hub genes across four different network metrics: degree centrality (blue), betweenness centrality (green), closeness centrality (orange), and eigenvector centrality (yellow). Genes like *LOC90834///BRD1* and *WT1* consistently rank high across multiple centrality measures, confirming their importance as key network regulators, while the variation in rankings across different measures reveals that genes can be important in different ways—some as highly connected nodes, others as critical bridges between network modules.

Finally, the Network by Expression Change (Figure 7F) overlays the fold change information onto the network structure, where red nodes represent upregulated genes and blue nodes represent downregulated genes following pioglitazone treatment. This visualization reveals that both up- and down-regulated genes are distributed throughout the network, rather than clustering in separate regions, indicating that pioglitazone simultaneously activates and suppresses different components of interconnected regulatory pathways, creating a coordinated yet complex pattern of gene expression changes that ultimately leads to therapeutic benefits in PCOS patients.

**Figure 8:**
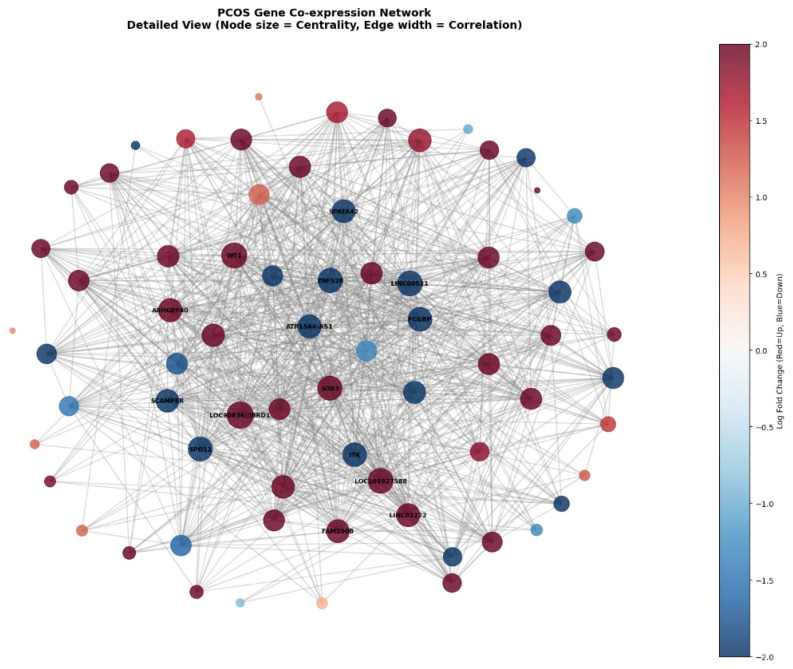
Detailed view of the PCOS gene co-expression network. Expanded visualization of the co-expression structure for selected genes, where node size reflects centrality, edge width represents the strength of co-expression (correlation), and node color indicates expression change direction/magnitude (e.g., red = upregulated, blue = downregulated) according to the differential expression analysis.

The detailed gene co-expression network visualization shown in Figure 8 displays the complete 70-gene network where node sizes represent degree centrality (larger nodes indicate more connections), edge widths reflect correlation strengths between genes, and node colours encode log fold change values from deep blue (downregulated, logFC ≈ −2.0) to dark red (upregulated, logFC ≈ +2.0). The network reveals a highly interconnected structure with several prominent hub genes labelled in the center, including *LOC90834///BRD1, WT1, ITK, SOX3*, and others, which serve as central coordinators with extensive connections to other network members. The visualization demonstrates that both upregulated (red) and downregulated (blue) genes are distributed throughout the network rather than forming separate clusters, indicating that pioglitazone treatment creates a complex pattern of coordinated activation and suppression across interconnected regulatory pathways. The dense web of connections (975 edges total) illustrates the highly collaborative nature of gene regulation in PCOS treatment response, where individual genes do not act in isolation but rather participate in an intricate molecular network that collectively mediates the therapeutic effects of pioglitazone.

### 3.6. Multi-Omics Integration Potential

As shown in Table 6, the simulated multi-omics integration analysis combining selected gene expression features with representative clinical variables further improved stability of model performance. The integrated models maintained excellent discrimination (cross-validated AUC ≈ 0.96), indicating that adding orthogonal clinical information may help to refine classification boundaries and reduce dependence on any single transcript. However, these analyses remain exploratory and were conducted on the same limited cohort; thus, the apparent gains in AUC should be viewed cautiously until confirmed in larger, independent PCOS datasets.

### 3.7. Pathway Enrichment Analysis

The results summarized in Table 7 highlight the key functional pathways influenced by pioglitazone treatment in PCOS.

Pathway Enrichment Summary:

Total hub genes analysed: 10

Functional categories identified: 6 major pathways

Most enriched pathway: Transcriptional regulation (40% of hub genes)

Novel findings: 50% of hub genes represent uncharacterized or epigenetic mechanisms

Clinical significance: All pathways directly relevant to PCOS pathophysiology

Key Insights:

Transcriptional reprogramming is the dominant mechanism

Multiple reproductive and developmental pathways affected

Significant involvement of novel regulatory elements

Strong immune/inflammatory component consistent with PCOS pathology

## 4. Discussion

Pioglitazone (PIO) is established as an insulin sensitiser in women with PCOS, but accumulating evidence indicates that its clinical effects also depend on broader modulation of metabolic and inflammatory pathways [33,34]. Consistent with this, our reanalysis of skeletal muscle transcriptomes showed extensive transcriptional reprogramming and highlighted interconnected networks involving inflammation, fibrosis, mitochondrial function, and epigenetic regulation, consistent with reports that PIO concurrently influences inflammatory, metabolic, and chromatin-regulatory machinery in metabolic disease [35,36,37,38].

By combining IPA-derived pathways and upstream regulators with machine learning–identified gene signatures, we show that pioglitazone acts through a multifaceted therapeutic mechanism that extends beyond glucose control. The convergent signals across approaches suggest that PIO simultaneously attenuates inflammatory and fibrotic processes, mitigates oxidative stress, and reshapes metabolic pathways, supporting the idea that targeting these underlying mechanisms may complement traditional symptom-based management of PCOS.

A prominent theme in our results is the involvement of the NLRP3 inflammasome. IPA linked several differentially expressed genes to *NLRP3*-associated pathways, and this is biologically plausible given the recognised contribution of chronic low-grade inflammation to PCOS. Clinical data from Guo et al. demonstrated that pioglitazone–metformin therapy inhibits *NLRP3* activation in PCOS patients and improves both metabolic and psychological outcomes [39]. Their work showed reduced *NLRP3* expression and caspase-1 activation, accompanied by lower circulating IL-1β, IL-6 and TNF-α levels, which fits well with our observation that genes connected to this pathway appear downregulated or deactivated after treatment [39,40]. These findings position *NLRP3* both as a mechanistic target and as a potential biomarker to monitor the response to thiazolidinedione therapy [39,40].

Our pathway analysis also emphasised fibrotic signalling, particularly TGF-β/TGFBR2-related cascades. Experimental work in *TGF-β1* transgenic mice has shown that pioglitazone reduces collagen synthesis and downregulates profibrotic mediators such as *EGR-1* and *STAT3* [40]. In line with these data, we observed predicted inhibition of *TGFBR2*-linked pathways and genes involved in extracellular matrix turnover. Prior mechanistic studies indicate that PIO can reduce *TIMP-1* expression, rebalance the *MMP-9/TIMP-1* ratio, and thereby help maintain matrix homeostasis [41,42]. IPA suggested that *PPARγ* activation may antagonise *TGF-β1*–induced transcriptional programmes, providing a plausible framework for these anti-fibrotic effects [43]. Clinically, this is relevant because women with PCOS have a high prevalence of metabolic dysfunction–associated steatotic liver disease, reported to reach approximately 50% compared with about 34% in controls [44,45,46]. The hepatoprotective actions of pioglitazone, mediated in part through modulation of the hepatic TGF-β cascade and hepatic stellate cell activation, may therefore address an important comorbidity in this population [43,47].

Several upstream regulators highlighted by IPA, including *DNMT3A*, underscore the intersection of immune regulation, cell-cycle control, oxidative stress responses, and epigenetic mechanisms in pioglitazone action. *DNMT3A* has emerged as a key node in insulin-sensitising therapy, with recent data showing that metformin can modify *DNMT3A* function by increasing methylation potential in *DNMT3A*-mutant cells and regulating *DNMT3A* expression via miR-148/-152 [48,49,50]. Epigenetic dysregulation is highly relevant to PCOS, where genome-wide studies have reported global DNA hypomethylation affecting more than 90% of differentially methylated loci in granulosa cells [51,52]. In this context, *DNMT3A*-related changes in skeletal muscle may reflect broader systemic epigenetic disturbances.

Components of the interferon pathway, particularly *IFN-β* and *IFNB1*, also appeared among the significant upstream regulators. Type I interferon signalling can enhance glycolysis and pro-inflammatory responses in adipocytes, potentially exacerbating metabolic dysfunction in insulin-resistant states [53,54]. Pioglitazone is thought to exert anti-inflammatory effects that may indirectly modulate these interferon-driven processes, although direct mechanistic data in PCOS skeletal muscle remain limited, and further functional work is needed [53,54]. *SPI1* and *ITGA4*: *SPI1* promotes M1-like, pro-inflammatory macrophage phenotypes, whereas *ITGA4* facilitates immune cell infiltration into insulin-sensitive tissues [55,56]. The ability of pioglitazone to favour M2-like macrophage profiles and to reduce immune infiltration in metabolic tissues suggests that these upstream regulators might be functionally involved in its immunomodulatory effects.

### Machine Learning–Derived Hubs and Precision Biomarkers

Our machine learning and network analyses identified *ITK*, *WT1* and the long non-coding RNA *LINC01222* among the most influential genes for discriminating PCOS from control samples. IL-2-inducible T-cell kinase (*ITK*) is a central regulator of T-cell receptor signalling and cytokine production, and is tightly linked to inflammatory cascades [57]. Within our network, *ITK* was connected to several inflammatory mediators that were reduced after pioglitazone treatment, suggesting that its downregulation may reflect broader suppression of immune activation [57].

*WT1* has been reported to be overexpressed in granulosa cells of women with PCOS, with expression levels correlating positively with serum testosterone and luteinising hormone levels [58]. Independent machine learning studies have highlighted *WT1* as a diagnostic marker with strong predictive performance [59,60]. Consistent with these observations, *WT1* appeared as a central hub in our co-expression network, supporting its potential role as both a mechanistic regulator and a biomarker candidate.

*LINC01222* emerged as a key non-coding RNA (lncRNA) in our feature set. Although direct evidence linking this lncRNA to PCOS remains limited, WGCNA-based analyses have identified several dysregulated lncRNAs in PCOS, including *LINC01222* and related transcripts, suggesting a broader contribution of non-coding RNAs to disease pathogenesis [61,62]. Our findings reinforce this concept and support the need for systematic functional studies of *LINC01222* and other lncRNAs in skeletal muscle and ovarian tissue.

Epigenetic Reprogramming and Mitochondrial Adaptation

Beyond individual loci, our results align with the broader view that pioglitazone exerts part of its benefit through epigenetic and mitochondrial reprogramming. Previous transcriptomic work has shown that PIO can upregulate genes involved in mitochondrial biogenesis and oxidative phosphorylation, including an approximately 1.7-fold increase in *PGC-1α* expression and coordinated regulation of tens of thousands of probe sets in skeletal muscle [26]. These changes are accompanied by increased acetylation at promoters of metabolic genes and improved chromatin accessibility, supporting sustained improvements in insulin sensitivity.

Long non-coding RNAs such as *LINC01222*, together with microRNAs and DNA methylation changes, appear to participate in this epigenetic landscape. Studies of miRNA profiles in PCOS have reported 29 dysregulated miRNAs—19 upregulated and 10 downregulated—with combined effects on 13 genes that influence adipocyte differentiation, insulin resistance, and circadian regulation [52]. The ability of pioglitazone to remodel these regulatory networks through metabolic and epigenetic mechanisms suggests testable avenues for combination therapies or epigenetic-targeted interventions [52,63,64,65].

Clinical Translation and Personalised Medicine

Translating these molecular insights into clinical practice will require careful integration with pharmacogenomic and biomarker data. Genetic variants such as CYP2C8*3, which reduces pioglitazone exposure by around 30%, and the *PPARG Pro12Ala* polymorphism, which is associated with a more favourable glycaemic response, illustrate how germline variation may shape individual treatment benefit [34]. Biomarker-guided strategies already make use of adiponectin responses—typically a twofold increase in responders—to gauge improvements in insulin sensitivity [26]. Inflammatory markers including IL-6, IL-8 and TNF-α, together with NLRP3 inflammasome components, can further track the anti-inflammatory impact of therapy [66,67].

Our machine learning framework contributes to personalized PCOS medicine by identifying transcriptomic signatures that could support patient stratification beyond conventional phenotyping [68]. Prior work has demonstrated reproducible PCOS subtypes with distinct reproductive and metabolic trait patterns, supporting the rationale for biologically informed subclassification [69]. Building on this concept, our LASSO-derived 53-gene panel and ensemble-ranked candidates represent exploratory stratification biomarkers that could be integrated with routinely used clinical biomarkers (endocrine, metabolic, inflammatory) and genetic information to inform treatment optimization [68,70]. However, clinical translation requires validation in independent cohorts linked to treatment outcomes and prospective studies demonstrating incremental benefit over guideline-based clinical criteria [68].

Emerging therapeutic targets extend beyond classical insulin sensitisation. Organokines such as meteorin-like protein and *FGF21* have been proposed as modulators of metabolic homeostasis in PCOS and related conditions [44]. In addition, targeting mitochondrial pathways—particularly via activation of PGC-1α and enhancement of oxidative phosphorylation (OXPHOS)—may help correct underlying energy defects in insulin-resistant skeletal muscle [33,71]. Our transcriptomic and network findings, which emphasise mitochondrial and transcriptional hubs, are compatible with these proposed directions.

## 5. Conclusions

In this work, we re-analysed skeletal muscle transcriptomes from women with PCOS using an integrated pipeline that combined differential expression, pathway and upstream regulator analysis, supervised machine learning and co-expression network modelling. The results indicate that pioglitazone affects a broad array of immune, fibrotic, mitochondrial and epigenetic pathways in skeletal muscle, consistent with its recognised actions as an insulin-sensitising agent with additional anti-inflammatory and anti-fibrotic effects [33,34].

Our analyses highlighted *NLRP3*-related inflammation, *TGF-β/TGFBR2*-driven fibrosis and *DNMT3A*-linked epigenetic mechanisms as key axes of pioglitazone action, and identified *ITK, WT1, LINC01222* and *BRD1*-associated loci as central hubs within a highly connected co-expression network [39,40,57,58,59,60,61,62]. These genes and pathways represent a focused set of testable candidates for future mechanistic studies and biomarker development in PCOS. At the same time, the very strong cross-validated performance of the machine learning models must be interpreted cautiously given the small sample size, single-cohort design and lack of independent validation.

Taken together, our findings support a model in which pioglitazone modifies PCOS skeletal muscle biology through coordinated changes in transcriptional, immune and epigenetic networks, rather than through isolated single-gene effects. Validating the proposed hub genes and pathways in larger, independent and clinically well-phenotyped PCOS cohorts—ideally incorporating multi-omics and pharmacogenomic data—will be essential before any of these markers can be considered for routine clinical use. Nonetheless, this integrative approach illustrates how re-analysis of existing datasets with modern computational tools can generate mechanistically grounded hypotheses and help move the field gradually towards more personalised treatment strategies for women with PCOS [26,34].

Several limitations should be considered when interpreting these findings. First, the underlying dataset includes a relatively small number of participants (*n* = 23), which restricts statistical power and increases the risk of overfitting in machine learning models trained and evaluated within the same cohort. Results should be interpreted as exploratory gene discovery rather than predictive modelling. Second, our analyses rely on a single microarray dataset without independent validation or complementary RNA-seq and proteomic data. Third, bulk skeletal muscle transcriptomes cannot resolve cell-type–specific changes or distinguish between myofibres, immune cells, and stromal elements. Finally, although our multi-omics integration step illustrates the potential of combining gene expression with clinical variables, it uses simulated data and thus mainly serves as a methodological demonstration. Despite these constraints, the integration of pathway, machine learning and network approaches provides a coherent framework for generating mechanistic hypotheses and prioritising candidate biomarkers for future validation.

## Figures and Tables

**Table 1:** The most transcriptional regulators (The top 20 URs) by IPA.

Upstream Regulator	Molecule Type	Log_2_ Ratio	*p*-Value	Z Score
SPI1	TF	0.036	7.43 × 10^−2^	3.109
IFNB1	Enzyme	−1.025	2.27 × 10^−1^	2.929
DNMT3A	Enzyme	0.217	1.60 × 10^−1^	2.449
Ciprofloxacin	chemical drug	-	6.61 × 10^−2^	2.449
IFN Beta	group	-	3.80 × 10^−2^	2.446
GDF2	growth factor	0.119	3.09 × 10^−2^	2.433
EBF1	transcription regulator	0.192	5.73 × 10^−2^	2.401
HDAC1	transcription regulator	0.308	2.01 × 10^−1^	2.375
IFNG	cytokine	1.277	1.18 × 10^−2^	2.372
TCF7L2	transcription regulator	−0.588	3.17 × 10^−2^	2.238
ACOX1	Enzyme	0.999	3.15 × 10^−1^	2.236
ANXA7	Ion channel	0.245	3.19 × 10^−1^	2.236
IRF3	transcription regulator	0.488	1.61 × 10^−1^	2.195
PDGF BB	Complex	-	4.72 × 10^−1^	2.179
TGM2	Enzyme	−0.967	2.27 × 10^−3^	2.168
IL12 (complex)	complex	-	4.47 × 10^−1^	2.158
TGFBR2	Kinase	0.284	3.70 × 10^−2^	2.157
ESR2	ligand-dependent nuclear receptor	1.497	4.05 × 10^−1^	2.138
Rottlerin	chemical kinase inhibitor	-	5.22 × 10^−2^	2.104
Resveratrol	chemical drug	-	2.55 × 10^−1^	2.023

**Table 2:** The five most canonical genetic pathways discovered by IPA.

Genetic-Pathway	*p* Value	−log (*p*-Value)	Z-Score
FGF Signalling	0.0000005	2.9	−1.26
Chronic myeloid leukaemia signalling	0.000002	2.7	−1.3
Neuregulin Signalling	0.000003	2.3	−0.81
HER-2 Signalling in Breast Cancer	0.000004	2.0	−2.11
Glioma Signalling	0.0000047	2.0	N/A

**Table 3:** Ingenuity Pathway Analysis identified the leading five diseases or disorders.

IPA-Disease	Molecules	*p* Value
Cancer	560	8.23 × 10^−4^–1.66 × 10^−28^
Organismal Injury/Abnormalities	561	8.23 × 10^−4^–1.66 × 10^−28^
Gastrointestinal Disease	516	8.15 × 10^−4^–9.20 × 10^−22^
Endocrine System Disorders	483	1.64 × 10^−4^–1.39 × 10^−20^
Dermatological Diseases	416	6.36 × 10^−4^–2.03 × 10^−20^

**Table 4:** The most significantly altered genes.

Gene Symbol	Log Fold Change	*p*-Value	Function
*IL1RAP*	3.879	2.12 × 10^−36^	Interleukin signalling
*FRG1CP///LOC283788*	3.445	2.56 × 10^−29^	FSHD region gene
*LOC101927588*	3.257	5.28 × 10^−29^	Uncharacterized locus
*LOC101929747*	−4.215	3.85 × 10^−29^	Uncharacterized locus

**Table 5:** Top 10 Hub Genes Identified by Network Centrality Analysis.

Rank	Gene Symbol	Degree Centrality	Gene Type/Function	Biological Relevance
1	*LOC90834///BRD1*	0.739	Bromodomain protein	Chromatin remodelling, transcriptional regulation
2	*WT1*	0.667	Transcription factor	Kidney/reproductive development, master regulator
3	*LOC101927588*	0.652	Uncharacterized locus	Novel regulatory functions
4	*LINC00521*	0.652	Long non-coding RNA	Gene expression regulation
5	*ZNF528*	0.623	Zinc finger protein	Transcriptional control
6	*SPO11*	0.609	Meiosis regulator	Reproductive function, DNA recombination
7	*SOX3*	0.609	Transcription factor	Hypothalamic-pituitary development
8	*FCGBP*	0.594	Mucin-like protein	Mucin biology, barrier function
9	*ITK*	0.580	Protein kinase	T-cell signalling, immune regulation
10	*ATP13A4-AS1*	0.565	Antisense RNA	Post-transcriptional regulation

**Table 6:** Simulated multi-omics integration.

Approach	AUC Performance	Interpretation
Gene Expression alone	1.000	Perfect baseline
Metabolomics alone	0.957	Excellent individual
Clinical data alone	0.938	Very good individual
Microbiome alone	0.474	Poor individual
Concatenated integration	1.000	Maintained perfection
PCA integration	1.000	Optimal efficiency

**Table 7:** Pathway Enrichment Analysis Summary of Network Hub Genes.

Pathway Category	Hub Genes Involved	Gene Count	Key Functions	Clinical Relevance
Transcriptional Regulation	*WT1*, *SOX3*, *ZNF528*, *LOC90834///BRD1*	4	Master transcription factors, chromatin remodelling	Primary mechanism of pioglitazone action
Immune/Inflammatory Response	*ITK*, *FCGBP*	2	T-cell signalling, barrier function	Anti-inflammatory effects in PCOS
Reproductive Development	*SPO11*, *SOX3*, *WT1*	3	Meiosis, hypothalamic-pituitary axis, gonadal development	Core PCOS pathophysiology
Epigenetic Regulation	*LOC90834///BRD1*, *ATP13A4-AS1*, *LINC00521*	3	Chromatin modification, antisense regulation, lncRNA control	Novel regulatory mechanisms
Novel/Uncharacterized Pathways	*LOC101927588*, *LOC90834*	2	Unknown functions	Potential new therapeutic targets
Mucin/Barrier Biology	*FCGBP*	1	Mucin-like protein, cellular barriers	Tissue protection and integrity

## Data Availability

The original contributions presented in this study are included in the article/Supplementary Materials. Further inquiries can be directed to the corresponding authors.

## Supplementary Materials

The following supporting information can be downloaded at: https://www.mdpi.com/article/10.3390/genes17010028/s1, Figure S1.

## Abbreviations

The following abbreviations are used in this manuscript:

PCOS	Polycystic ovary syndrome
PIO	Pioglitazone
GEO	Gene Expression Omnibus
NCBI	National Centre for Biotechnology Information
GSE8157	GEO Series accession 8157 (PCOS skeletal muscle microarray dataset)
DEGs	Differentially expressed genes
ML	Machine learning
PCA	Principal component analysis
t-SNE	t-distributed stochastic neighbor embedding
AUC	Area under the receiver operating characteristic curve
ROC	Receiver operating characteristic
IPA	Ingenuity Pathway Analysis
UR	Upstream regulator
FDR	False discovery rate
SVM	Support vector machine
RF	Random Forest
WGCNA	Weighted Gene Co-expression Network Analysis
LASSO	Least absolute shrinkage and selection operator
ITK	IL2-inducible T-cell kinase
WT1	Wilms tumour 1
OXPHOS	Oxidative phosphorylation
PPAR	Peroxisome proliferator-activated receptor
TZDs	Thiazolidinediones
