# Supplementary figures and images for: Integrative Machine Learning and Network Analysis of Skeletal Muscle Transcriptomes Identifies Candidate Pioglitazone-Responsive Biomarkers in Polycystic Ovary Syndrome
Source: Genes (Basel). 2025 Dec 29;17(1):28. doi: 10.3390/genes17010028 (PMC12841334; doi:10.3390/genes17010028)

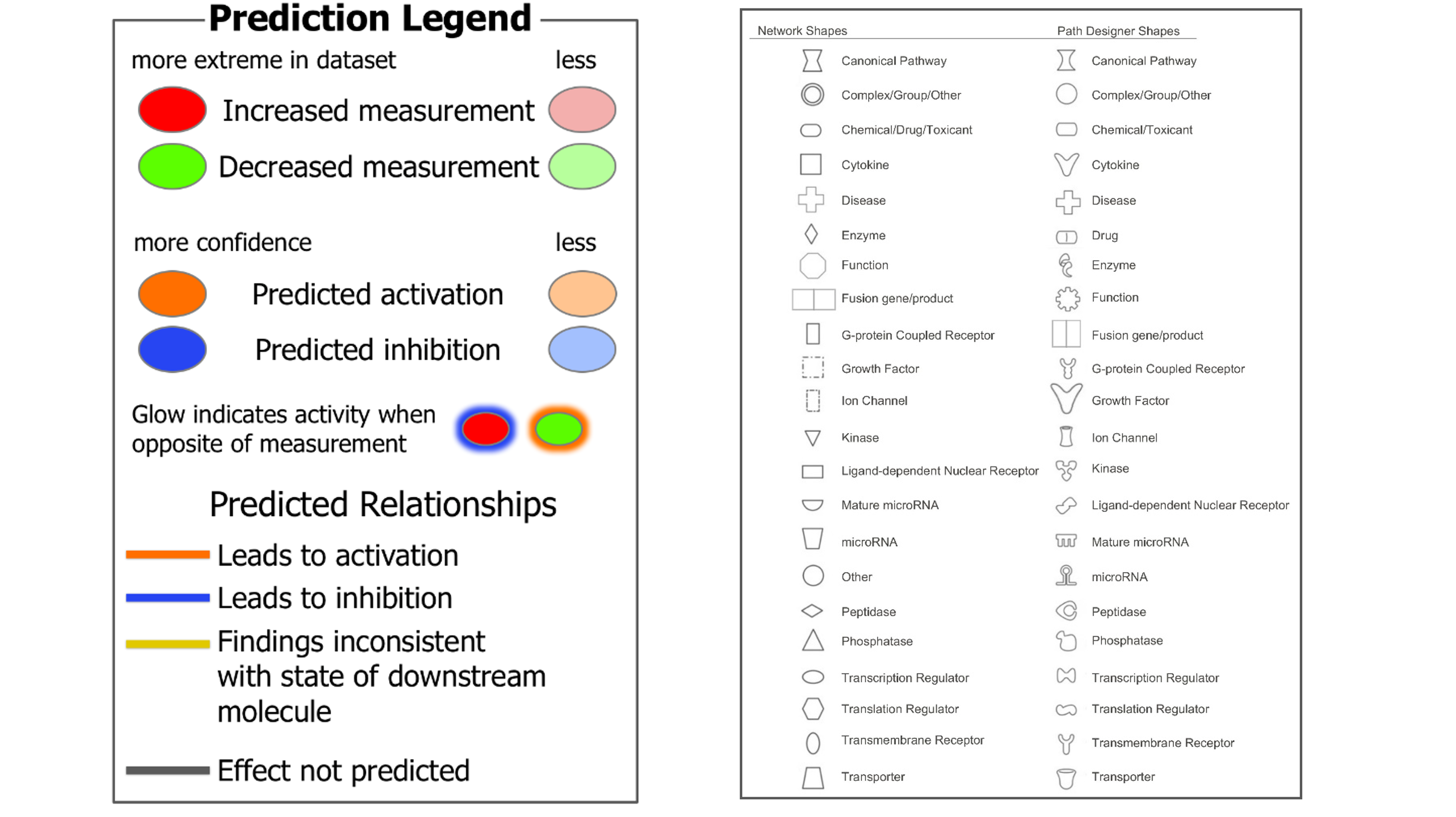

Supplement: Supplementary file 1 [file genes-17-00028-s001.zip › genes-4030730-supplementary.png]
